# Exploring the Effects of Variety and Amount of Mindfulness Practices on Depression, Anxiety, and Stress Symptoms: Longitudinal Study on a Mental Health–Focused eHealth System for Patients With Breast or Prostate Cancer

**DOI:** 10.2196/57415

**Published:** 2024-11-21

**Authors:** Francesca Malandrone, Sara Urru, Paola Berchialla, Pierre Gilbert Rossini, Francesco Oliva, Silvia Bianchi, Manuel Ottaviano, Sergio Gonzalez-Martinez, Vladimir Carli, Gaetano Valenza, Enzo Pasquale Scilingo, Sara Carletto, Luca Ostacoli

**Affiliations:** 1 Department of Clinical and Biological Sciences, University of Turin, Regione Gonzole 10, Orbassano, 10043, Italy, 39 0116334200; 2 Department of Cardiac, Thoracic, Vascular Sciences and Public Health, Unit of Biostatistics, University of Padova, Padova, Italy; 3 Clinical Psychology Unit, University Hospital Città della Salute e della Scienza di Torino, Turin, Italy; 4 School of Engineering, Research Center, University of Pisa, Pisa, Italy; 5 Life Supporting Technologies, Universidad Politécnica de Madrid, Madrid, Spain; 6 Department of Learning, Informatics, Management and Ethics, National Centre for Suicide Research and Prevention of Mental Ill-Health, Karolinska Institute, Stockholm, Sweden; 7 Department of Psychology, University of Turin, Turin, Italy; 8 Psychology Unit, ASL TO5, Turin, Italy

**Keywords:** depression, anxiety, stress, internet-based, mental health, mindfulness, breast cancer, prostate cancer, cancer-related mental distress, emotional distress, psychological distress, mindfulness-based interventions, MBI, e-MBI, dispositional mindfulness, self-compassion, mental wellbeing, mobile phone

## Abstract

**Background:**

Patients with cancer often face depression and anxiety, and mindfulness-based interventions, including internet-based versions, can effectively reduce these symptoms and improve their quality of life. This study aims to investigate the impact of internet-based mindfulness-based interventions (e-MBIs) on anxiety, depression, and stress symptoms in patients with prostate or breast cancer.

**Objective:**

The primary aims are to assess the association between the amount and variety of e-MBI practices and symptom reduction. Second, this study aims to examine how baseline information such as sociodemographic characteristics, dispositional mindfulness (DM), and dispositional self-compassion (DSC) correlate with both app usage and symptom reduction.

**Methods:**

Participants included 107 patients with cancer (68 women with breast cancer and 38 men with prostate cancer) enrolled in a hospital setting. They were assigned to the intervention group of the NEVERMIND project, using the e-BMI module via the NEVERMIND app. A longitudinal design involved Pearson correlation analysis to determine the relationship between the amount and duration of e-MBI practices. Linear regression analysis was conducted to gauge the dose-response effect, evaluating the impact of DM and DSC on depression, anxiety, and stress. Negative binomial regression was conudcted to study sociodemographic factors’ influence on the amount of practice in e-MBIs.

**Results:**

The participants with more diverse and sustained mindfulness practices experienced significant reductions in depression, anxiety, and stress. A high correlation (0.94) between e-MBI practices and symptom reduction was also highlighted. Male, married, and highly educated patients were more likely to engage in mindfulness. Even if DM and DSC did not impact the amount or variety of practices correlated, they were correlated with symptom reduction, showing that higher levels were associated with significant reductions in depression, anxiety, and stress.

**Conclusions:**

While more e-MBI practice is linked to reduced anxiety, depression, and stress, this study emphasizes the crucial role of variety of practice over amount. DM and DSC are key in shaping intervention effectiveness and may act as protectors against psychological distress. Using app log data, our research provides a unique perspective on e-MBI impact, contributing to cancer care understanding and guiding future studies.

## Introduction

Patients with cancer often experience psychological problems such as depression and anxiety, which can exacerbate the challenges of their treatment and management [1]. Due to their prevalence, breast cancer in women and prostate cancer in men are the types of cancer most extensively studied in terms of their psychological consequences. Literature reports that both sexual and emotional functions are significantly affected in patients with these cancers, leading to repercussions on quality of life, mental health, and the quality of social relationships [2,3]. These issues can negatively impact treatment compliance, hospitalization, response, and prognosis, and ultimately, the survival rate [4-6]. Research has shown that interventions to reduce distress (such as depression and anxiety) can have a positive downstream impact on patients, families, cancer outcomes, and the medical system [7,8].

Mindfulness-based interventions (MBIs) have emerged as effective approaches for reducing anxiety and depressive symptoms and for enhancing the quality of life among patients with cancer [9-12]. The duration of these interventions varied across studies but typically entailed weekly sessions for 5-8 weeks. Notably, certain studies have suggested that the extent of improvement in mindfulness skills is associated with greater reductions in psychological distress following the intervention, highlighting the potential importance of the amount of mindfulness training provided. In the oncological context, the efficacy of MBIs has mainly been studied in patients with breast cancer [12-14]. Together with prostate cancer, they are the most prevalent cancer types studied for women and men, respectively [15]. Despite the higher prevalence of these cancers, distinctions between breast, prostate, and other types of cancer are not typically emphasized when considering symptoms such as anxiety, depression, and stress. These psychological issues do not seem to be specific to any particular type of cancer; therefore, most studies and systematic reviews generally address these symptoms uniformly across various cancer types without differentiation. Beyond the oncological context, although the optimal dosage of MBIs required to alleviate psychological symptoms remains unclear, reviews have demonstrated that even low-dose and brief MBIs can yield mental health benefits and improve self-regulation [16-18]. Recent meta-analyses have further indicated that short mindfulness exercises, when administered as stand-alone programs without extensive introductory or discussion components, exhibit superior effectiveness in reducing anxiety and depression compared to control conditions [19]. Nevertheless, it is generally observed that increased mindfulness instruction and practice (ie, higher amount) are associated with improved outcomes, as these factors represent crucial elements of MBIs [20-22]. A more recent meta-analysis conducted by Strohmaier [23] revealed that while no significant dose-response relationship was found for psychological outcomes such as depression, anxiety, or stress, a significant association was observed between amount of mindfulness practice and the improvement of mindfulness skills.

Several studies have examined the relationship between baseline characteristics and the efficacy of MBIs, identifying certain outcome predictors. For instance, higher levels of baseline depression have been associated with increased treatment response for anxiety, whereas lower levels of baseline self-compassion predicted enhanced treatment response for depression [24]. Similarly, another study found that a strong fear of recurrence or low levels of conscientiousness could reduce adherence [25]. Consistent with this, another study reported that better program adherence, reflected in participants’ higher attendance to the MBI program, was a significant predictor of both remission and response [26]. Additionally, self-compassion has been shown to negatively correlate with depression, anxiety, and stress in both clinical [27,28] and nonclinical [29-31] populations. To the best of our knowledge, existing studies primarily focus on the impact of mindfulness on symptom reduction. This study is the first to investigate the moderation of intervention effects, highlighting the novelty of our research.

Internet-based or smartphone-delivered mindfulness-based interventions (e-MBIs) demonstrated effectiveness in supporting mental health and reducing psychological symptoms in various populations, including those with depression, anxiety, and organic diseases [32-38]. In particular, they have shown efficacy in reducing depression, stress, and anxiety not only in patients with breast or prostate cancer [39-42] but also across most other types of cancer [24,43-45]. e-MBIs offer a feasible and flexible option for cancer survivors, providing advantages such as easy accessibility, anonymity, 24/7 availability, reduced reliance on trained therapists, cost-effectiveness, and time-saving benefits [18,46]. Recent systematic reviews and meta-analyses have highlighted the feasibility and efficacy of e-MBIs in improving several outcomes—such as anxiety, depression, posttraumatic stress disorder, fatigue, pain—suggesting their potential superiority over traditional face-to-face interventions [18,47-49]. Although attrition rates may be higher in e-MBIs compared to face-to-face programs [50], e-MBIs have shown to be cost-effective and accessible [44] promoting positive changes in subjective levels of stress, anxiety, depression, fatigue, sleep problems, mindfulness, posttraumatic growth, pain, and general health indicators [18,47-49]. However, the existing literature supporting this type of intervention is currently limited and of medium to low quality.

The primary objective of this study was to investigate how different use of an e-MBI delivered via an e-Health system impacts anxiety, depression, and stress levels in patients with prostate or breast cancer. We aim to (1) examine the relationship between the amount of e-MBI practices completed by participants and the observed reduction in symptoms to test the hypothesis that a greater practice amount is associated with a stronger reduction in symptoms of anxiety, depression, and stress; and (2) to evaluate the effect of practising a greater variety of e-MBI exercises, that is, a greater amount of more types of practices, on symptoms, to test our hypothesis that a greater assortment of practices may be associated with a greater improvements in symptoms of anxiety, depression, and stress.

Furthermore, this study sought to address the following secondary research objectives: (3) to identify possible predictors of the intensity of use of the e-MBI modules among variables collected at baseline, that is, sociodemographic characteristics, dispositional mindfulness (DM) level, and dispositional self-compassion (DSC) level. Our hypothesis was that higher mindfulness and self-compassion scores may be associated with the amount of practice; (4) to investigate about a potential association between preintervention DM and DSC levels and the reduction of symptoms. This analysis aims to determine whether these individual features are linked to symptom improvement. We hypothesized that higher DM and DSC may be associated with greater symptom reduction.

## Methods

### Overview of the NEVERMIND System

This study used data from the NEVERMIND European Union–funded Horizon 2020 project. The NEVERMIND system is a comprehensive solution designed to address depressive symptoms in patients with somatic illnesses (ie, myocardial infarction, breast cancer, prostate cancer, kidney failure, or lower limb amputation). It consists of two main components: a sensorized shirt and a mobile app. The shirt collects physiological data, while the app gathers mental health questionnaires, allowing the system to predict depressive symptom levels and to deliver tailored care based on depressive symptom severity. This approach ensured personalized support for participants at varying levels of need, ultimately aiming to improve their well-being. No mental health indicators resulted in positive feedback, while early signs prompted lifestyle advice in the form of different modules within the app (exercise, sleep hygiene, and dietary recommendations) and an e-MBI. Severe symptoms activated online cognitive behavioral therapy. A detailed description of the design, content, and functionality of the NEVERMIND system has been published in previous publications [51,52]. In an randomized controlled trial involving 425 patients with severe somatic illnesses, such as breast cancer, prostate cancer, myocardial infarction, kidney failure, or leg amputation, the NEVERMIND system was found to be superior to standard care in reducing depressive symptoms [52].

### Study Design

This study used a longitudinal design to explore the effect of the NEVERMIND e-MBI module on symptoms of depression, anxiety, and stress among patients with breast or prostate cancer. The NEVERMIND trial was registered in the German Clinical Trials Register (DRKS00013391).

### Ethical Considerations

This study was approved by the Ethical Committee of the Città della Salute e della Scienza di Torino University Hospital, Torino, Italy (CS2/11), and the Ethical Committee of the San Luigi Gonzaga University Hospital, Orbassano, Italy (185/2015). The original institutional review board approvals covered secondary data analysis without requiring additional consent. All data were anonymized, and no compensation was provided to participants.

### Participants

Participants included in this study were patients with breast or prostate cancer from the intervention group of the NEVERMIND project, recruited from the Piedmont Oncological Network at San Luigi Gonzaga University Hospital, Turin, Italy, and the Breast Unit-Oncology Department and Urology Department at Città della Salute e della Scienza University Hospital, Turin, Italy, from November 2017 to December 2019. The inclusion and exclusion criteria for the NEVERMIND trial are included in the published protocol [51].

The inclusion criteria for the subsample in this study encompassed patients with breast or prostate cancer assigned to the NEVERMIND intervention group who completed this study and engaged in at least one e-MBI practice lasting a minimum of 5 minutes. Exclusion criteria involved individuals assigned to the control group in the NEVERMIND study, patients in the NEVERMIND intervention group diagnosed with other severe somatic conditions (eg, kidney failure, leg amputation, and myocardial infarction), and patients in the NEVERMIND intervention group who dropped out of this study before receiving the NEVERMIND system.

### Data Collection

#### Overview

Participants were asked to complete a series of questionnaires at baseline to assess demographic information, symptoms of anxiety, depression, and stress, and levels of DM and DSC. Afterward, participants were informed about how the NEVERMIND system works and were instructed to use the system for a period of 12 weeks, committing to using the app almost every day. Therefore, participants were assessed at the end of the 12-week period of use, which included questionnaires regarding symptoms of anxiety, depression, and stress. A description of each variable used in this study is provided in the following sections.

#### Demographic and Clinical Variables

Sociodemographic data collected for patients recruited in this study included age, gender, education level (dichotomized into low, ie, below high school diploma or degree, and high, ie, degree or higher), marital status, employment, and living status (ie, cohabitant or alone).

Depression symptoms were measured using the Beck Depression Inventory II (BDI-II) scale [53]. BDI-II is a 21-item self-report instrument assessing the presence and severity of depressive symptoms in the past week. The total score (ie, the sum of all items) indicates the level of depression within a range from 0 to 63, with higher scores indicating higher levels of depressive symptoms.

Anxiety and stress symptoms were measured using the anxiety and the stress subscale, respectively, of the Depression Anxiety and Stress Scale (DASS-21) [54]. DASS-21 consists of 21 items, 7 for each of 3 subscales (ie, depression, anxiety, and stress). Each item scored from 0 to 3. The total is then doubled to align with the full version of the DASS-21, leading to a possible score range from 0 to 42 for each subscale, with higher scores indicating higher levels of symptoms. In accordance with the methodology outlined by Carli et al [52], the BDI-II was selected as the primary outcome measure, while the DASS-21 depression subscale was administered only at baseline (T0), following the principle of data minimization.

The Mindful Attention Awareness Scale (MAAS) [55] was used to assess DM, that is, open or receptive awareness and attention to what is happening in the present with a nonjudgmental attitude. The MAAS is a 15-item self-report questionnaire of mindfulness measured on a 6-point Likert scale ranging from 1=“almost always” to 6=“almost never.” Higher scores represent greater DM.

Self-compassion was measured with the Self-Compassion Scale-Short Form [56]. It is a 12-item self-report questionnaire on a 5-point Likert scale ranging from 1=“almost never” to 5=“almost always,” assessing how often people behave kindly and caringly toward themselves in difficult life situations. The final Self-Compassion Scale-Short Form score ranges from 12 to 70. A higher score indicates a higher level of self-compassion.

#### e-MBI Amount and Variety of Practices

Data regarding the number, duration, and type of e-MBI practices were extracted from the usage log of the NEVERMIND app. The NEVERMIND e-MBI module included different types of mindfulness practices of different durations (5, 10, or 20 min), taken from the mindfulness-based stress reduction protocol [57,58], the Compassion-Focused Therapy protocol [59-61] and the Four Immeasurable of the Buddhist tradition [62-64]. Table S1 in Multimedia Appendix 1 shows all the practices covered by this module. Each day, the app proposed a recommended practice to the user, based on the preferences expressed by the patient at the time of registration and the mental health symptoms detected by the questionnaires embedded in the app. Completion of the mindfulness practices unlocked access to other types of practices, granting the patient an incremental path over the period of use of the NEVERMIND system (12 wk). For each patient, the total number, type and duration of e-MBI practices used during the 12 weeks of use of the NEVERMIND system were calculated. Since the amount of practices performed by the patients was associated with the variety of practices unlocked by the app incrementally, we have provided in Table S2 of the Multimedia Appendix 1 the number of patients per group for each practice type. Additionally, to further delineate this distinction between practice dosage and variety across groups, we introduced an indicator variable named “time×practice” (TP). This variable partitions patients into 2 distinct categories: those who exclusively engaged in the initial practices recommended by the app, encompassing “sensorial opening,” “body scan 1,” “body scan 2,” “enriching listening to nature,” and “loving presence” (TP=0), and those who embraced a broader array of practices, incorporating both a greater number and variety over successive weeks (TP=1).

### Statistical Analysis

Descriptive statistics were reported as mean and SD for continuous variables and frequency and percentage for categorical ones. Kendall correlation coefficient was used to assess the correlation between the number of e-MBI practices used by the patients and the amount of time spent practicing. Given the high correlation between the number of e-MBI practices and their total duration (*r*=0.88, 95% CI 0.83 to 0.91, *P*<.001), the number of practices was used as independent variable in a linear regression analysis evaluating the dose-response effect on depressive, anxiety, and stress symptoms, adjusting for baseline levels. Further, two linear regressions to evaluate the dose-response effect considering the number of practices and adjusting for baseline values of depressive, anxiety, or stress symptoms were performed separately for patients with breast or prostate cancer. Descriptive statistics about the type of practices were reported including the total number of practices and the number of patients for each type of practice. Linear regression was applied to assess the influence of being in TP=0 and TP=1 groups on symptom reduction (ie, depression, anxiety, and stress). This analysis was also performed separately for patients with breast or prostate cancer. Negative binomial regression was performed to estimate the influence of the sociodemographic factors and baseline characteristics on the count of completed e-MBI practices. The 2 groups, defined on indicator variable TP, were compared for sociodemographic characteristics, psychological features, and psychopathology using the chi-square test for categorical variables and Wilcoxon-Mann-Whitney test for continuous ones. Further, linear regression was applied to evaluate the impact of DM and DSC on psychopathology (ie, depression, anxiety, or stress). All the statistical analyses were performed with the statistical software R (version 4.1.2; The R Foundation).

## Results

Out of the initial cohort of 129 patients with breast or prostate cancer assigned to the NEVERMIND intervention group, 107 individuals actively engaged in mindfulness practices for at least 5 minutes and constituted this study’s sample. Table 1 presents the baseline characteristics of this cohort.

**Figure 1.**
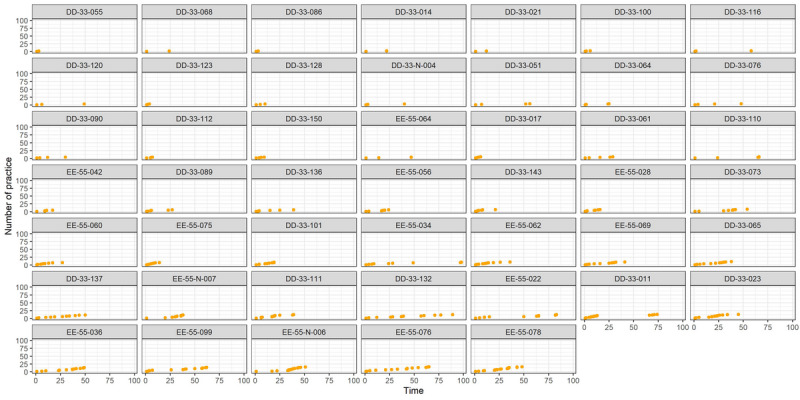
Practice trajectories for TP=0 group patients. The practice trajectories of each patient in the TP=0 group are reported. the horizontal axis shows the number of days of practice, while the vertical axis shows the cumulative number of practices. TP: time×practice (indicator variable used in this study).

**Figure 2.**
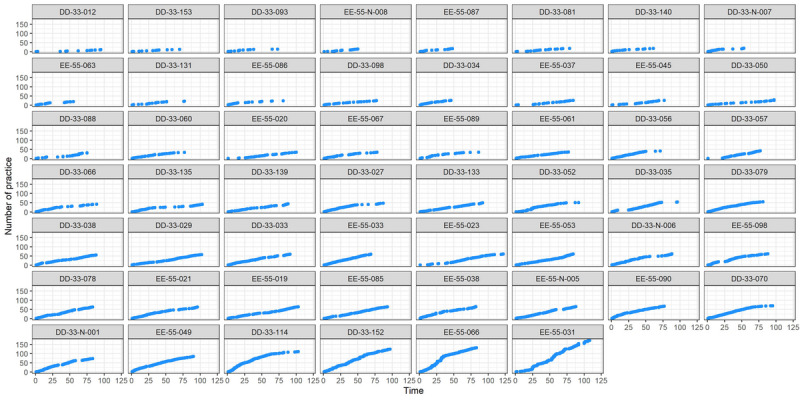
Practice trajectories for TP=0 group patients. The practice trajectories of each patient in the TP=1 group are reported. The horizontal axis shows the number of days of practice, while the vertical axis shows the cumulative number of practices. TP: time×practice (indicator variable used in this study).

As illustrated in Figures 1 and 2, patients in TP=0 (n=53) engaged in mindfulness practices less frequently, mainly during the initial period. Conversely, patients in TP=1 (n=54) exhibited more frequent, diverse, and sustained practices over an extended duration. Patients in TP=1 demonstrated a more comprehensive exploration of various practice types, maintaining consistency over time.

**Table 1.** Sociodemographic characteristics of patients at baseline.

	Values
**Sex, n (%)**	
Female	68 (63.6)
Male	39 (36.4)
**Education, n (%)**	
Low	29 (27.1)
High	78 (72.9)
**Marital status, n (%)**	
Single	92 (86)
Married	15 (14)
**Employment status, n (%)**	
Unemployed	22 (20.6)
Employed	85 (79.4)
**Living status, n (%)**	
Cohabitant	56 (52.3)
Alone	51 (47.7)
Age, mean (SD)	60.16 (9.30)
BDI^a^ score, mean (SD)	12.54 (9.44)
DASS-21-D^b^ score, mean (SD)	4.48 (4.64)
DASS-21-A^c^ score, mean (SD)	3.03 (3.22)
DASS-21-S^d^ score, mean (SD)	6.50 (4.79)
MAAS^e^ score, mean (SD)	4.43 (0.86)
SCS-SF^f^ score, mean (SD)	3.22 (0.46)

a BDI: Beck Depression Inventory.

b DASS-D: Depression Anxiety and Stress Scale-Depression.

c DASS-A: Depression Anxiety and Stress Scale-Anxiety.

d DASS-S: Depression Anxiety and Stress Scale-Stress.

e MAAS: Mindful Attention Awareness Scale.

f SCS-SF: Self-Compassion Scale-Short Form.

The results of the dose-response effect adjusted for baseline values are reported in Table 2: a significant slight improvement of depression, anxiety, and stress symptoms with the increase of the number of practices was observed. In Multimedia Appendix 1, further analyses are provided, considering patients with breast or prostate cancer separately. Among the breast cancer cohort, the number of e-MBI practices significantly reduced depressive, anxiety, and stress symptoms (Table S3 in Multimedia Appendix 1). Conversely, no significant reductions were observed in the prostate cancer cohort (Table S4 in Multimedia Appendix 1).

Regarding the effect of a greater variety of e-MBI practices on the reduction of symptoms, patients in group TP=1 had statistically significant lower level of depressive, anxiety, and stress symptoms than patients in group TP=0, despite the adjustment for baseline score (Table 3).

**Table 2.** Results of linear regression analysis evaluating the dose-response effect of the number of practices on depressive, anxiety, and stress symptoms, adjusted for baseline levels.

Outcomes and predictors		Estimates	95% CI	*R* ^2^	AIC^a^	*P* value
**BDI^b^**				0.34	706.13	—^c^
	Baseline	0.48	0.34 to 0.62	—	—	<.001
	Of practices	–0.06	–0.10 to –0.01	—	—	.01
**DASS-A^d^**				0.34	499.27	—
	Baseline	0.53	0.37 to 0.69	—	—	<.001
	Of practices	–0.02	–0.04 to 0.00	—	—	.02
**DASS-S^e^**				0.44	555.65	—
	Baseline	0.58	0.44 to 0.72	—	—	<.001
	Of practices	–0.03	–0.06 to –0.01	—	—	.002

a AIC: Akaike information criterion.

b BDI: Beck Depression Inventory.

c Not applicable.

d DASS-A: Depression Anxiety and Stress Scale-Anxiety.

e DASS-S: Depression Anxiety and Stress Scale-Stress.

**Table 3.** Results of linear regression analysis evaluating the effect of TP groups on depression, anxiety, and stress, adjusted for baseline levels.

Outcomes and predictors		Estimates	95% CI	*R* ^2^	AIC^a^	*P* value
**BDI^b^**				0.35	704.29	—^c^
	Baseline	–3.87	–6.49 to –1.25	—	—	.004
	TP^d^=1	0.47	0.33 to 0.61	—	—	<.001
**DASS-A^e^**				0.37	494.73	—
	Baseline	–1.64	–2.63 to –0.64	—	—	.001
	TP=1	0.55	0.39 to 0.70	—	—	<.001
**DASS-S^f^**				0.44	556.82	—
	Baseline	–2.02	–3.36 to –0.69	—	—	.003
	TP=1	0.58	0.44 to 0.72	—	—	<.001

a AIC: Akaike information criterion.

b BDI: Beck Depression Inventory.

c Not applicable.

d TP: time×practice (indicator variable used in this study).

e DASS-A: Depression Anxiety and Stress Scale-Anxiety.

f DASS-S: Depression Anxiety and Stress Scale-Stress.

Tables S5 and S6 in Multimedia Appendix 1 report the impact of TP groups on depression, anxiety, and stress in patients with breast or prostate cancer, respectively. The results indicate that in the breast cancer cohort, patients who engaged in a higher number of e-MBI practices (TP=1) experienced significant reductions in symptoms (Table S5 in Multimedia Appendix 1), whereas this effect was not seen in the prostate cancer cohort (Table S6 in Multimedia Appendix 1). Table S7 in Multimedia Appendix 1 compares the number of practices and baseline symptom values across the cancer cohorts. The analysis revealed that males (ie, the prostate cancer cohort) engaged in more e-MBI practices than females, as indicated by the negative binomial regression model in Table 4. However, despite this higher engagement, significant symptom reduction was observed only in the breast cancer cohort, likely due to their higher baseline symptom values compared to patients with prostate cancer.

**Table 4.** Results of negative binomial regression analysis evaluating the impact of sociodemographic factors and baseline characteristics on the amount of practices.

Predictors^a^	IRR^b^ (95% CI)	*P* value
Sex: Male	1.85 (1.12 to 3.07)	.01
Age	1.00 (0.97 to 1.03)	.82
Employment: employed	0.86 (0.55 to 1.36)	.51
Marital status: married	2.94 (1.47 to 5.57)	.001
Living arrangement: alone	2.19 (0.92 to 5.12)	.06
Education: high	2.02 (1.19 to 3.34)	.007
DASS-A^c^	0.97 (0.90 to 1.05)	.53
DASS-S^d^	0.99 (0.93 to 1.06)	.81
BDI^e^	1.03 (0.99 to 1.06)	.13
MAAS^f^	0.97 (0.71 to 1.30)	.82
SCS-SF^g^	1.16 (0.75 to 1.81)	.51

a *R*2=0.37; Akaike information criterion=931.35.

b IRR: internal rate of return.

c DASS-A: Depression Anxiety and Stress Scale-Anxiety.

d DASS-S: Depression Anxiety and Stress Scale-Stress.

e BDI: Beck Depression Inventory.

f MAAS: Mindful Attention Awareness Scale.

g SCS-SF: Self-Compassion Scale-Short Form.

Table S8 in Multimedia Appendix 1 presented descriptive statistics concerning the types of e-MBI practices, encompassing the total practice count and the number of patients for each practice type. Additionally, Table S9 in Multimedia Appendix 1 detailed the sociodemographic and baseline characteristics of the two groups, categorized according to the variable TP. As regards variables associated with the amount of e-MBI practices, results show that male, married, and highly educated patients were more prone to be engaged in mindfulness practices (Table 4).

This result was corroborated by a statistically significant difference in the number of highly educated patients that emerged between the TP=0 and TP=1 groups, showing that highly educated individuals practiced more extensively and with greater variety (Table S9 in Multimedia Appendix 1). Regarding the influence of DM and DSC on the use of e-MBI modules, no association was found between TP=1 group and both DM and DSC (Table S9 in Multimedia Appendix 1).

Finally, this study aimed to explore the potential correlation between preintervention DM and DSC levels and their respective contributions to symptom reduction. Increasing levels of MAAS were associated with a statistically significant reduction in depression, anxiety, and stress. Similarly, higher values of SC were linked to lower levels of depressive, anxiety, and stress symptoms (Table S10 in Multimedia Appendix 1).

## Discussion

### Principal Findings

To the best of our knowledge, this study contributed to the existing literature by examining the association between sociodemographic factors, mindfulness practice characteristics, and psychological symptoms reduction in patients with cancer. This provides a unique perspective considering these variables within the oncological context, addressing a crucial gap in current research. While patients with cancer often encounter psychological challenges impacting treatment outcomes, e-MBIs have shown effectiveness in reducing cancer-related psychological distress, offering flexibility and cost-effectiveness [24,43-45]. Nevertheless, the available literature remains limited in both quality and quantity.

The first aim of our study was to investigate whether the amount of e-MBI practice is related to symptom reduction. Confirming our hypothesis, results indicate that a greater practice dose was associated with a more pronounced reduction in anxiety, depression, and stress symptoms. These results are interesting although the observed reduction in symptoms is relatively modest from a clinical point of view, which is probably related to the baseline low levels of anxiety, depression, and stress symptoms. This was particularly evident when we separated male and female (and thus, patients with breast or prostate cancer). Differences in symptoms at baseline, in fact, appear to play a role in showing posttreatment improvement, despite men having practiced more. Specifically, within the breast cancer cohort, the number of e-MBI practices significantly reduced depressive, anxiety, and stress symptoms even though males in the prostate cancer cohort engaged in more practices than females. However, symptom reduction was evident only in the breast cancer cohort, likely due to their notably higher baseline values compared to patients with prostate cancer. The efficacy of MBIs in reducing depression and anxiety symptoms in patients with cancer has been emphasized by two recent meta-analyses [9,44]. A recent study showed that e-MBIs can be as effective as in-person MBIs, with significant psychological improvements when provided to health care workers [65]. Regarding the influence of practice amount on symptoms, the current literature remains limited and unclear, especially within the oncologic field and when investigating e-MBIs. While some studies and reviews have identified a positive link between the amount of MBI practice and symptom reduction [20-22,66], others have indicated no correlation between practice quantity and symptom alleviation. Strohmaier and colleagues [23,67] found no evidence that larger doses are more beneficial than smaller doses for predicting psychological outcomes, aligning with some previous research in nonclinical populations. Similarly, a review by Cillessen and colleagues [47] on MBIs for psychological and physical health outcomes in patients and survivors of cancer found that the measured intervention dose in hours does not appear to be a significant mediator of the effect. The second aim was to investigate how practising a more diverse range of e-MBI exercises, encompassing both the quantity and types of practices, impacts the reduction of symptoms. Our results show that a greater variety of practices, rather than quantity per se, was associated with more substantial reductions in symptoms related to anxiety, depression, and stress. As far as we know, this is the first study to have investigated the variety of mindfulness practices’ effect on psychological symptoms. Few studies, mainly on nonclinical samples, have investigated the effects of specific mindfulness practices highlighting their differences and similarities in psychological outcomes [68-70] and from a neurobiological point of view [71-73]. For example, Carmody and Baer [74] showed that varying practice times for different meditation exercises, such as sitting meditation, body scan, and mindful yoga, are associated with distinct outcomes, indicating the specificity of each practice in addressing different aspects of psychological health within MBIs. Similarly, Sauer-Zavala et al [75] explored various meditation practices’ impact on well-being. Mindful yoga showed greater psychological well-being improvement, and both mindful yoga and sitting meditation were more effective in enhancing emotion regulation compared to body scan. Zeng and colleagues [76] found that different subtypes of focused attention meditation, such as appreciative joy meditation and compassion meditation, had distinct effects on positive emotions in recent laboratory studies. Considering the early stage of research in this field, it is only possible to speculate that our results may be explained by the notion that a greater variety of practices enables the targeting of different psychological abilities and resources, thereby contributing to the reduction of symptoms.

Having explored the impact of both the amount and variety of mindfulness practices on symptoms and confirming the effective reduction of symptoms, this study delved into the examination of baseline factors. Specifically, it investigated how sociodemographic characteristics, DM, and DSC at baseline influenced the amount and variety of practice, namely the use of the e-MBI module.

Examining the categories displaying a greater inclination to engage in practice, we found that males, married individuals, and those with higher levels of education exhibited a heightened propensity. Within the oncologic field, the evaluation of sociodemographic characteristics influencing the use of MBIs, and the reduction of psychological symptoms remains limited. Existing literature [47,77,78] lacks adequate evidence to evaluate the relationship with age, gender, and education. In a recent study investigating the perceived usability of the NEVERMIND system among patients with breast or prostate cancer, Petros et al [79] highlighted the potential influence of gender differences on the use and efficacy of eHealth interventions. Notably, their findings suggested that, despite sociodemographic variations, a digital divide did not significantly impact the usability of the NEVERMIND system. That study found that women exhibited higher favorability toward this eHealth intervention, while men demonstrated an increased usage of it. Comparable results were identified in studies beyond the cancer context; for instance, Olano and colleagues [80] explored the impact of sociodemographic factors on mindfulness practices, discovering that higher education correlated with increased engagement, while men were half as likely as women to participate.

Regarding the relationship between preintervention DM and DSC levels and participants’ engagement with the mindfulness module, surprisingly, we found that these two dimensions did not impact the use of MBI practices, influencing neither the amount nor variety of practices but solely contributing to the reduction of symptoms. This suggests that DM and DSC could make individuals more receptive to even a lower dose of treatment, serving as protective and facilitating factors that amplify intervention effects. Conversely, low DM may act as a barrier, diminishing intervention impact. This hypothesis finds partial support in the outcomes pertaining to the fourth aim of this study. The exploration of the correlation between preintervention levels of DM and DSC with their respective impacts on symptom reduction yielded interesting findings. Specifically, the results indicated a positive association, whereby higher DM correlated with more pronounced reductions in symptoms of depression, anxiety, and stress, while increased DSC enhanced stress reduction. Despite limited literature on the moderating role of DM in psychological intervention efficacy, these results align with prior research. Elevated DM was found to have a buffering effect on anxiety, depression, and stress symptoms [81]. Similarly, a longitudinal study demonstrated that DSC benefited patients with cancer, leading to reduced symptoms of depression, anxiety, and fatigue over time [82]. Both DM and DSC appeared advantageous for psychological well-being, inversely related to depressive symptoms and negative affect [83]. In a recent systematic review, Tomlinson and colleagues [84] highlighted an inverse relationship between DM and pain catastrophizing in the nonclinical population, suggesting DM’s potential to enhance individuals’ resilience and act as a buffer against the development of negative thought patterns predictive of psychological distress.

### Limitations and Strengths of This Study

This study has some limitations. First, it primarily focused on self-help mindfulness interventions, potentially limiting the generalizability of our findings from practitioner-delivered mindfulness programs. Additionally, while app logs provided precise data on the quantity and type of mindfulness practice, the absence of data on individual mindfulness practices hindered our ability to assess the relative impact of different practices on symptom modification. We relied predominantly on demographic data to analyze factors facilitating or obstructing intervention effectiveness, without access to other psychological variables that could enhance our understanding of this aspect. Furthermore, our study exclusively examined two specific cancer types, which may restrict the applicability of our results to a broader range of cancer diagnoses. On the other hand, to the best of our knowledge, this is the first study to benefit from objective data, obtained from app logs, regarding the quantity and type of mindfulness practice, as opposed to relying solely on patient self-reports as is common in most studies. This approach increased the accuracy and reliability of our findings in this specific aspect of the research.

### Research and Clinical Implications

This study’s clinical and scientific implications underscore the need for expanded research within the clinical population, particularly regarding e-MBIs for patients with cancer. The findings provide a foundation for structuring e-MBIs in clinical contexts to optimize outcomes with a favorable cost or benefit ratio, emphasizing the importance of tailoring interventions based on individual preferences. Further exploration of mindfulness practices’ impact on psychological symptoms across various clinical populations is needed, promoting a more comprehensive understanding of their potential benefits. The data-driven insights from app logs open avenues for future research to delve deeper into refining interventions, leveraging technology, and gathering more diverse and detailed data to enhance the overall effectiveness of e-MBI in clinical settings.

### Conclusions

In conclusion, we found that greater practice amount of e-MBIs was associated with notable reductions in anxiety, depression, and stress symptoms, aligning with previous literature on the positive effects of MBIs. Additionally, the variety of mindfulness practices, rather than their amount, exhibited a significant impact on symptom reduction, underscoring the multifaceted nature of mindfulness. DM and DSC emerged as influential factors in shaping the effectiveness of these interventions, further highlighting their potential role as facilitators and protectors against psychological distress. Despite the limitations, our study introduces a unique perspective by using objective data from app logs to better understand the impact of e-MBIs on the psychological well-being of patients with cancer. Our findings emphasize the importance of mindfulness in addressing the psychological challenges faced by patients with cancer and offer insights into the factors that can enhance or hinder intervention effectiveness. This research contributes to the broader understanding of the role of e-MBI in cancer care and provides a foundation for future studies in this field.

## Supplementary material

10.2196/57415 Multimedia Appendix 1 Additional tables and analyses which provide further details on mindfulness practices, patient engagement, and the statistical evaluation of practice effects on psychological symptoms, along with baseline characteristics and the influence of dispositional factors.

## Abbreviations

BDI-II: Beck Depression Inventory II
DASS-21: Depression Anxiety and Stress Scale
DM: dispositional mindfulness
DSC: dispositional self-compassion
e-MBI: internet-based mindfulness-based intervention
MAAS: Mindful Attention Awareness Scale
MBI: mindfulness-based intervention
TP: time×practice (indicator variable used in this study)
