# Supplementary material for: Exploring the Effects of Variety and Amount of Mindfulness Practices on Depression, Anxiety, and Stress Symptoms: Longitudinal Study on a Mental Health–Focused eHealth System for Patients With Breast or Prostate Cancer
Source: JMIR Ment Health. 2024 Nov 21;11:e57415. doi: 10.2196/57415 (PMC11604212; doi:10.2196/57415)
Supplement: Multimedia Appendix 1 [file mental-v11-e57415-s001.docx]

**Table S1.** Mindfulness practices in the e-MBI module of the NEVERMIND system.

| **Practice name** | **Categories** | **Reference** |
| --- | --- | --- |
| Body scan 1 | Body Scan | Kabat-Zinn, 1990 |
| Sensorial opening | Body Scan, Equanimity | Kabat-Zinn, 1990; Kraus & Sears, 2009 |
| Enriching listening to nature | Loving Kindness, Appreciative Joy | Hofmann et al., 2011; Royzman & Rozin, 2006 |
| Body scan 2 | Body Scan | Kabat-Zinn, 1990 |
| Loving presence | Loving Kindness, Appreciative Joy | Kurtz & Martin, 2019 |
| Breathing 1 | Breathing Meditation | Lutz et al., 2008 |
| Yoga lying | Body Awareness | Kabat-Zinn, 1990 |
| Gratitude to the body | Loving Kindness, Compassion | Hofmann et al., 2011; Neff, 2003; Gilbert, 2010; Rinpoche, 2015 |
| Yoga standing | Body Awareness | Kabat-Zinn, 1990 |
| Loving-kindness | Loving Kindness | Hofmann et al., 2011 |
| Breathing 2 | Breathing Meditation | Lutz et al., 2008 |
| Exploring and welcoming emotions in the body | Loving Kindness,  Body Awareness | Hofmann et al., 2011; Kabat-Zinn, 1990 |
| Mountain meditation | Equanimity | Kraus & Sears, 2009 |
| Self-compassion | Compassion | Neff, 2003; Gilbert, 2010; Rinpoche, 2015 |
| Loving-kindness 2 | Loving Kindness | Hofmann et al., 2011 |
| Awareness of breathing, sounds and thoughts 1 | Breathing Meditation, Equanimity | Lutz et al., 2008; Kraus & Sears, 2009 |
| Light flow | Compassion | Neff, 2003; Gilbert, 2010; Rinpoche, 2015 |
| Compassion | Compassion | Neff, 2003; Gilbert, 2010; Rinpoche, 2015 |
| Awareness of breathing, sounds and thoughts 2 | Breathing Meditation, Equanimity | Lutz et al., 2008; Kraus & Sears, 2009 |
| Tonglen | Compassion | Neff, 2003; Gilbert, 2010; Rinpoche, 2015 |
| Awareness of breath with grounding and alignment | Breathing Meditation | Lutz et al., 2008 |
| Breathing 3 | Breathing Meditation | Lutz et al., 2008 |

**Table S2.** Number of patients who practised at least once the following practice types stratified by the TP groups.

| **Practice name** | **TP=0** | **TP=1** |
| --- | --- | --- |
| Body scan 1 | 52 | 54 |
| Sensorial opening | 46 | 54 |
| Enriching listening to nature | 21 | 54 |
| Body scan 2 | 19 | 53 |
| Loving presence | 4 | 52 |
| Breathing 1 | 0 | 33 |
| Awareness of breathing, sounds and thoughts 1 | 0 | 4 |
| Awareness of breath with grounding and alignment | 0 | 8 |
| Awareness of breathing, sounds and thoughts 2 | 0 | 11 |
| Breathing 2 | 0 | 17 |
| Breathing 3 | 0 | 53 |
| Compassion | 0 | 27 |
| Self-compassion | 0 | 26 |
| Exploring and welcoming emotions in the body | 0 | 30 |
| Gratitude to the body | 0 | 46 |
| Light flow | 0 | 18 |
| Loving kindness 1 | 0 | 25 |
| Loving kindness 2 | 0 | 33 |
| Mountain meditation | 0 | 30 |
| Tonglen | 0 | 18 |
| Yoga lying | 0 | 45 |
| Yoga standing | 0 | 40 |

**Table S3.** Results of linear regression to evaluate the dose-response effect considering the number of practices and adjusting for baseline values of depressive, anxiety, and stress symptoms in breast cancer patients.

| Outcome | R2 | AIC | Predictors | Estimates | 95%CI | *p* |
| --- | --- | --- | --- | --- | --- | --- |
| BDI | 0.34 | 706.13 | Baseline | 0.48 | 0.34 – 0.62 | <.001 |
|  |  |  | N. of practices | -0.06 | -0.10 – -0.01 | .01 |
| DASS-A | 0.34 | 499.27 | Baseline | 0.53 | 0.37 – 0.69 | <.001 |
|  |  |  | N. of practices | -0.02 | -0.04 – -0.00 | .02 |
| DASS-S | 0.44 | 555.65 | Baseline | 0.58 | 0.44 – 0.72 | <.001 |
|  |  |  | N. of practices | -0.03 | -0.06 – -0.01 | .002 |

**Table S4.** Results of linear regression to evaluate the dose-response effect considering the number of practices and adjusting for baseline values of depressive, anxiety, and stress symptoms in prostate cancer patients.

| Outcome | R2 | AIC | Predictors | Estimates | 95%CI | *p* |
| --- | --- | --- | --- | --- | --- | --- |
| BDI | 0.19 | 260.16 | Baseline | 0.53 | 0.15 – 0.92 | .008 |
|  |  |  | N. of practices | -0.01 | -0.07 – 0.05 | .69 |
| DASS-A | 0.11 | 193.57 | Baseline | 0.53 | -0.01 – 1.07 | .06 |
|  |  |  | N. of practices | -0.01 | -0.04 – 0.01 | .40 |
| DASS-S | 0.32 | 201.37 | Baseline | 0.51 | 0.25 – 0.77 | <.001 |
|  |  |  | N. of practices | -0.01 | -0.03 – 0.02 | .60 |

**Table S5.** Results of linear regression to evaluate the effect of TP groups on depression, anxiety, and stress in breast cancer patients.

| Outcome | R2 | AIC | Predictors | Estimates | 95%CI | *p* |
| --- | --- | --- | --- | --- | --- | --- |
| BDI | 0.36 | 451.3 | Baseline | 0.47 | 0.29 – 0.65 | <.001 |
|  |  |  | TP=1 | -4.82 | -8.33 – -1.31 | .008 |
| DASS-A | 0.43 | 309.55 | Baseline | 0.55 | 0.37 – 0.72 | <.001 |
|  |  |  | TP=1 | -1.91 | -3.17 – -0.66 | .003 |
| DASS-S | 0.42 | 357.27 | Baseline | 0.57 | 0.39 – 0.76 | <.001 |
|  |  |  | TP=1 | -2.80 | -4.61 – -0.99 | .003 |

**Table S6.** Results of linear regression to evaluate the effect of TP groups on depression, anxiety, and stress in prostate cancer patients.

| Outcome | R2 | AIC | Predictors | Estimates | 95%CI | *p* |
| --- | --- | --- | --- | --- | --- | --- |
| BDI | 0.21 | 258.92 | Baseline | 0.56 | 0.17 – 0.94 | .005 |
|  |  |  | TP=1 | -2.36 | -6.51 – 1.80 | .26 |
| DASS-A | 0.14 | 192.52 | Baseline | 0.54 | 0.01 – 1.08 | .046 |
|  |  |  | TP=1 | -1.15 | -2.92 – 0.63 | .20 |
| DASS-S | 0.32 | 201.38 | Baseline | 0.51 | 0.26 – 0.77 | <.001 |
|  |  |  | TP=1 | -0.51 | -2.50 – 1.48 | .61 |

**Table S7.** Number of practices and baseline values of depressive, anxiety, and stress symptoms across breast and prostate cancer cohorts. Wilcoxon test was applied to test the difference between the cohorts.

|  | Breast cancer | Prostate cancer | *p* |
| --- | --- | --- | --- |
| N. of practices | 23.35 (26.75) | 37.18 (35.95) | .03 |
| BDI | 15.62 (9.88) | 7.18 (5.44) | <.001 |
| DASS-A | 3.81 (3.63) | 1.67 (1.66) | .001 |
| DASS-S | 7.74 (4.87) | 4.36 (3.85) | <.001 |

**Table S8.** Number of patients per practice type.

| **Practice name** | **n (%)** | **mean (sd)** |
| --- | --- | --- |
| Body scan 1 | 54 (100.0) | 4.76 (2.99) |
| Sensorial opening | 54 (100.0) | 4.39 (2.89) |
| Enriching listening to nature | 54 (100.0) | 4.20 (2.29) |
| Body scan 2 | 53 (98.1) | 3.66 (2.31) |
| Loving presence | 52 (96.3) | 3.50 (2.21) |
| Breathing 1 | 53 (98.1) | 3.53 (2.15) |
| Yoga lying | 45 (83.3) | 3.40 (1.54) |
| Gratitude to the body | 46 (85.2) | 3.37 (1.45) |
| Yoga standing | 40 (74.1) | 3.28 (1.65) |
| Loving-kindness | 33 (61.1) | 3.48 (1.97) |
| Breathing 2 | 33 (61.1) | 3.58 (1.41) |
| Exploring and welcoming emotions in the body | 30 (55.6) | 3.33 (1.21) |
| Mountain meditation | 30 (55.6) | 3.00 (1.14) |
| Self-compassion | 26 (48.1) | 3.31 (1.72) |
| Loving-kindness 2 | 25 (46.3) | 3.60 (1.50) |
| Awareness of breathing, sounds and thoughts 1 | 17 (31.5) | 3.41 (0.94) |
| Light flow | 18 (33.3) | 4.17 (2.60) |
| Compassion | 27 (50.0) | 3.26 (1.53) |
| Awareness of breathing, sounds and thoughts 2 | 11 (20.4) | 4.18 (2.18) |
| Tonglen | 18 (33.3) | 3.28 (0.89) |
| Awareness of breath with grounding and alignment | 8 (14.8) | 3.00 (1.51) |
| Breathing 3 | 4 (7.4) | 4.75 (4.19) |

**Table S9.** Differences of baseline characteristics between the TP groups.

|  |  | TP=0 (53) | TP=1 (54) | *p* |
| --- | --- | --- | --- | --- |
| Age |  | 61.00 [54.00, 67.00] | 62.00 [54.25, 66.75] | .88 |
| Sex | Female | 37 (69.8) | 31 (57.4) | .23 |
|  | Male | 16 (30.2) | 23 (42.6) |  |
| Education | Low | 16 (30.2) | 6 (11.1) | .02 |
|  | High | 37 (69.8) | 48 (88.9) |  |
| Marital status | Single | 19 (35.8) | 10 (18.5) | .05 |
|  | Married | 34 (64.2) | 44 (81.5) |  |
| Employment_status | Unemployed | 29 (54.7) | 27 (50.0) | .70 |
|  | Employed | 24 (45.3) | 27 (50.0) |  |
| Living arrangement | Cohabitant | 46 (86.8) | 46 (85.2) | .99 |
|  | Alone | 7 (13.2) | 8 (14.8) |  |
| BDI |  | 10.00 [6.00, 17.00] | 10.00 [6.25, 15.75] | .81 |
| DASS-D |  | 4.00 [1.00, 7.00] | 3.00 [1.00, 6.00] | .84 |
| DASS-A |  | 2.00 [0.00, 4.00] | 2.00 [1.00, 4.00] | .89 |
| DASS-S |  | 6.00 [2.00, 9.00] | 7.00 [1.25, 9.00] | .99 |
| MAAS |  | 4.60 [3.73, 5.13] | 4.47 [3.77, 5.07] | .96 |
| SC |  | 3.33 [2.92, 3.58] | 3.17 [2.92, 3.56] | .70 |

**Table S10.** Results of linear regression to evaluate the impact of dispositional mindfulness and self-compassion on depression, anxiety, and stress considering the change over time of MAAS and SC and adjusting for the baseline values of symptoms and MAAS and SC, respectively.

|  | BDI | | | DASS-A | | | DASS-S | | |
| --- | --- | --- | --- | --- | --- | --- | --- | --- | --- |
| Predictors | Est | CI | *p* | Est | CI | *P* | Est | CI | *p* |
| Baseline | 0.45 | 0.29 – 0.61 | *<.001* | 0.45 | 0.29 – 0.62 | *<.001* | 0.43 | 0.28 – 0.59 | <.001 |
| MAAS Baseline | -2.54 | -4.49 – -0.59 | *.01* | -1.22 | -1.91 – -0.53 | *.001* | -2.12 | -3.08 – -1.16 | <.001 |
| MAAS Change | -5.75 | -7.55 – -3.95 | *<.001* | -2.05 | -2.75 – -1.34 | *<.001* | -2.95 | -3.88 – -2.02 | <.001 |
| R^2^ | 0.51 | | | 0.48 | | | 0.57 | | |
| AIC | 665.94 | | | 473.04 | | | 526.48 | | |
| Baseline | 0.42 | 0.27 – 0.57 | *<.001* | 0.50 | 0.33 – 0.66 | *<.001* | 0.56 | 0.41 – 0.70 | <.001 |
| SC Baseline | -3.51 | -6.88 – -0.14 | *.04* | -1.14 | -2.38 – 0.09 | *.07* | -1.34 | -3.05 – 0.37 | .12 |
| SC Change | -3.53 | -6.25 – -0.81 | *.01* | -1.37 | -2.41 – -0.33 | *.01* | -2.03 | -3.39 – -0.67 | .004 |
| R^2^ | 0.35 | | | 0.35 | | | 0.44 | | |
| AIC | 707.19 | | | 499.75 | | | 558.88 | | |
